# Case Study: Soft tissue infection with *Raoultella ornithinolytica*

**DOI:** 10.1016/j.jpra.2022.04.004

**Published:** 2022-05-01

**Authors:** DT Goodman, D Murphy, J Dorairaj

**Keywords:** Paronychia, Microbiology, Infectious diseases, Hand and upper limb, Raoultella ornithinolytica

## Abstract

*Raoultella ornithinolytica* is a rare encapsulated Gram-negative aerobic and facultative anaerobic rod belonging to the Enterobacteriaceae family. It tends to inhabit water and soil environments and can be found on insects, fish, ticks, and termites, but can also found in the hospital environment.^1^^,^^2^*R ornithinolytica* has been documented in respiratory, urinary, gastrointestinal, and biliary tract infections as well as bacteraemia and systemic infections but has rarely been documented in soft tissue infections.^2^ This case study describes a recurrent paronychia infection secondary to *R ornithinolytica* in a young woman not responding to antibiotics and successfully treated with surgical management.

## Case presentation

A 42-year-old right hand dominant typist with a past medical history of hypertension, Hashimoto thyroiditis and Raynaud's syndrome presented to the emergency department following a referral by her GP with a 4-month history of recurring infection in her right middle finger. The patient presented with mild swelling, erythema and fluctuating pain. The patient had been treated with four courses of antibiotics including flucloxacillin, co-amoxiclav and fusidic acid cream by her GP with little relief. She was a non-smoker. On examination, she had a dry yellow eschar with underlying pus at the radial aspect of her distal middle finger with swelling and erythema of the area surrounding her fingernail. On clinical examination she was tender with reduced range of movement at the distal interphalangeal joint. She was systemically well and apyrexial.

## Investigations

A pain film radiograph was performed which showed no osteomyelitic changes. No blood tests were performed.

## Treatment

The patient underwent partial excision of the nail plate to facilitate debridement and curettage of the granulation tissue under local anesthetic. A thorough washout of the wound with 0.9% saline and 50/50 mix of hydrogen peroxide and saline was performed. Intraoperative swabs were taken and sent for culture and sensitivity. The patient was discharged the same day on a seven-day course of oral flucloxacillin.

## Follow-up

The patient was reviewed four days post operatively in our clinic. Her wound was clean and dry with no signs of lingering infection. She was non-tender and had full range of movement at her DIPJ. Her cultures had returned the result of *R. ornithinolytica* and *Citrobacter* spp. both of which were resistant to flucloxacillin, doxycycline and clindamycin but sensitive to ciprofloxacin. The patient was given a subsequent phone call consultation and she reported a return of her symptoms in the same finger following the procedure. She had received further treatment with flucloxacillin from her primary care physician which again only partially treated the infection, after which the patient's GP was contacted and advised on the culture and sensitivities.

## Discussion

Chronic paronychia is defined as inflammation of the perionychium for more than 6 weeks with multiple causes including colonization with fungal or bacterial pathogens and recurrent contact with moisture or chemical irritants.^3^ Common symptoms at presentation include erythema, pain and swelling however these symptoms are often not as intense as with presentations of acute paronychia with episodic exacerbations more common in chronic paronychia following exposure to moist environments.^4^ Nail changes associated with chronic paronychia include ridging, grooving, discolouration and rounding of the nail plate. The differential diagnosis of acute/chronic paronychia should include herpes simplex infection (Herpetic whitlow) and malignancies such as squamous cell carcinoma, melanoma and Karposi sarcoma among others. Management of paronychia includes topical or systemic antibiotics followed by surgical management for refractory cases.^3^

*Raoultella spp*. are non-motile encapsulated Gram-negative bacilli commonly found in the natural environment and were previously thought to be less pathogenic. However, a subsequent literature review conducted by Seng et al. (2016) demonstrated that this species is more frequently being isolated in cases of nosocomial infections, as well as cases in immunocompromised hosts.^2^^,^ ^5^ Its pathogenicity is due to its ability to form biofilms, polysaccharide capsules, siderophores and fimbriae as well as the presence of the chromosomal *bla* gene which has been postulated to give it resistance to beta-lactam antibiotics including penicillins.^5^^,^ ^6^ This species has been documented as sensitive to treatment with cefotaxime, ciprofloxacin and gentamicin among others.^7^

There are three species of *Raoultella* including *R. terrigena, R. planticola* and *R. ornithinolytica* however these were initially classified under the genus *Klebsiella* until advanced phylogenetic testing which employed 16S rRNA and rpoB sequence analysis allowed us to differentiate *Raoultella* from *Klebsiella* thus creating the genus *Raoultella* in 2001.^1^ *Klebsiella ornithinolytica* was first identified in 1989.^6^A search of the literature from the 1980′s to 2006 shows only four documented cases of human *Raoultella* infections, however this has risen to more than 130 overall cases to the present day.^7^

*R. ornithinolytica* is a rare cause of infection in humans and a review by Seng et al. (2016) documented 112 cases of *R. ornithinolytica* infections, but only 7 (6%) of these were isolated following community-acquired wound and skin infections.^2^ This same review noted that 25% of the patients were immunodeficient with 25% of this group diagnosed with concurrent malignancy and 22% diagnosed with diabetes. Subsequently, Ayoade et al. (2018) documented a case of polymicrobial breast wound infection with associated fat necrosis which included *R. ornithinolytica* resistant to ampicillin and trimethoprim/sulfamethoxazole ^8^. No further cases of *R. ornithinolytica* soft tissue infections have been documented in the literature since.

The mortality associated with *Raoultella spp*. ranges from 8% to 100% depending on the number of subjects per case series in the literature. Higher mortality is associated more with *R. planticola* compared to *R. ornithinolytica*, and more so with associated bacteraemia and sepsis.^8^ Seng et al. described just one case of mortality associated with a skin infection.^2^

Our patient had a polymicrobial infection with *R ornithinolytica* and *Citrobacter* spp. and on further discussion with the patient we could not identify the source of her infection. She did not possess any of the risk factors that would make her more susceptible to this bacterium such as immunodeficiency, malignancy, diabetes mellitus, solid organ transplant or long-term antibiotic therapy.^9^^,^ ^10^ Nor had she recently been an inpatient and undergone invasive procedures that Seng et al. describe as being linked to nosocomial infections such as urinary catheterisation, mechanical ventilation or port catheter placement.^2^

## Conclusion

The purpose of this case report is to document a rare case of multidrug-resistant R. ornithinolytica in an immunocompetent 42-year-old woman that was partially treated with empirical antibiotics in the community followed by surgical washout and debridement and finally with targeted antimicrobial therapy. It highlights the importance of culture and sensitivity investigations during an incision and drainage procedure and follow-up with the correct antibiotic therapy for a potentially atypical bacterial infection.

## Declaration of Competing Interest

None declared.
